# The Relationship between Bulk Silicone and Benzophenone-Initiated Hydrogel Coating Properties

**DOI:** 10.3390/polym10050534

**Published:** 2018-05-16

**Authors:** Damla Keskin, Taraneh Mokabbar, Yutao Pei, Patrick van Rijn

**Affiliations:** 1 University Medical Center Groningen, Department of Biomedical Engineering-FB40, W.J. Kolff Institute for Biomedical Engineering and Materials Science-FB41, University of Groningen, A. Deusinglaan 1, 9713 AV Groningen, The Netherlands; d.keskin@umcg.nl; 2 Engineering and Technology Institute Groningen, University of Groningen, Nijenborgh 4, 9747 AG Groningen, The Netherlands; t.mokabber@rug.nl (T.M.); y.pei@rug.nl (Y.P.); 3 Zernike Institute for Advanced Materials, University of Groningen, Nijenborgh 4, 9747 AG Groningen, The Netherlands

**Keywords:** benzophenone, coatings, hydrogel, PDMS, UV-mediated free radical polymerization, mechanical properties

## Abstract

Polydimethylsiloxane (PDMS) is a silicone elastomer-based material that is used in various applications, including coatings, tubing, microfluidics, and medical implants. PDMS has been modified with hydrogel coatings to prevent fouling, which can be done through UV-mediated free radical polymerization using benzophenone. However, to the best of our knowledge, the properties of hydrogel coatings and their influence on the bulk properties of PDMS under various preparation conditions, such as the type and concentration of monomers, and UV treatment time, have never been investigated. Acrylate-based monomers were used to perform free radical polymerization on PDMS surfaces under various reaction conditions. This approach provides insights into the relationship between the hydrogel coating and bulk properties of PDMS. Altering the UV polymerization time and the monomer concentration resulted in different morphologies with different roughness and thickness of the hydrogel coating, as well as differences in the bulk material stiffness. The surface morphology of the coated PDMS was characterized by AFM. The cross section and thickness of the coatings were examined using scanning electron microscopy coupled with energy-dispersive X-ray spectroscopy. The dependence of coating development on the monomer type and concentration used was evaluated by surface hydrophilicity, as measured by water contact angle. Elongation-until-break analysis revealed that specific reaction conditions affected the bulk properties and made the coated PDMS brittle. Therefore, boundary conditions have been identified to enable high quality hydrogel coating formation without affecting the bulk properties of the material.

## 1. Introduction

In recent years, silicone rubber (polydimethylsiloxane, PDMS) has gained growing importance due to its beneficial properties in many promising applications including insulating coatings, microfluidics, optics, tubing in both food industry and biomedical devices (catheters), and implants [1,2,3,4,5,6,7,8]. PDMS is a silicone elastomer, which is a transparent, chemically inert, non-toxic, and biocompatible material with good mechanical properties [9,10]. However, PDMS lacks many of the desired characteristics for these applications because of its hydrophobic nature; for instance, PDMS shows undesirable fouling behavior by different organisms and wettability complications in microfluidic systems [2,11,12,13]. Moreover, the hydrophobic surface causes undesired adsorption such as proteins, which therefore affects the function of the material [14,15,16]. To overcome these negative issues of PDMS, many different types of surface modification approaches have been developed. Previously, polyethylene oxide (PEO) and polyethylene glycol (PEG)-based coatings [17,18,19], UV-ozone treatments [20], silanization, oxygen plasma treatments [21,22], and zwitterion-based coatings [23,24] were applied to enhance the hydrophilicity of PDMS surfaces. Nevertheless, the long-term stability of these surface modifications still needs to be developed further [2,25]. On the other hand, polymer brushes have offered many fascinating possibilities to enhance the fouling resistance of PDMS, but so far, this method still requires complex synthetic approaches and the usage of undesired Fe or Cu catalysts [26,27,28,29].

Easy coating approaches have been developed for hydrogel coatings, which offer interesting possibilities as they are easy to functionalize, adaptive and deformable, and can be made responsive towards external stimuli [30,31]. Moreover, surface-attached hydrogel coatings are very easy to fabricate and can be implemented under various conditions. Further, the resulting hydrogel layer is stable under many conditions [32]. An approach to create hydrogel coatings on a PDMS surface is via UV-mediated free radical polymerization using benzophenone (BP). A large surface area is readily modified by photo-initiated polymerization because these reactions can be performed under mild experimental conditions [33]. In particular, UV-mediated photo-grafting using benzophenone is a well-established procedure that does not require special surface functionalization before polymerization, other than the infusion of PDMS with benzophenone [34,35]. UV-excitation is used to create surface-bound free radicals as benzophenone excited and by proton abstraction, creates a methyl-radical [36], thereby initiating the free-radical reaction where the surface confined methyl radicals react with the monomers ((meth)acrylates) present in solution [37,38]. This approach is used for different applications within coloring, medical coatings, and adhesives [39,40,41]. However, neither the bulk properties of PDMS after applying these coatings nor the effect of possible changes in these bulk properties on optimum coating properties were considered previously.

**Figure 1:**
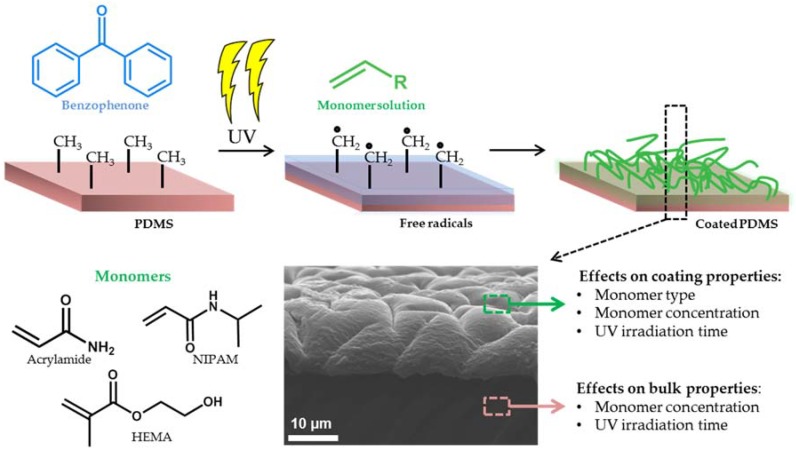
Schematic illustration of UV-mediated free radical polymerization onto a polydimethylsiloxane PDMS surface. The molecular structures used, a representative image for a hydrogel coating, and the main parameters that affect either the coating or the bulk properties are shown.

In this paper, we use benzophenone UV-mediated free radical polymerization to demonstrate the relationship between coating conditions and hydrogel coating properties, and we relate these findings to the bulk properties of the modified PDMS. Hydrogel coatings were formed using *N*-Isopropylacrylamide (NIPAM), hydroxyethylmethacrylate (HEMA) and acrylamide (AAm). These monomers exhibit hydrophilic behavior and have been used frequently to enhance characteristics of PDMS surfaces [28,42,43]. The influence of monomer type, monomer concentration, and UV irradiation time on the coating characteristics and bulk properties were investigated and optimized. The properties of these covalently-bound coatings were studied by determining the water contact angle (WCA), surface morphology, coating thickness, and elongation-until-break analysis. To the best of our knowledge, the coating preparation conditions related to coating characteristics and bulk material properties, which provide key insights in applying hydrogels coatings without losing functional material properties, have not been reported. Therefore, we have performed mechanical tests to understand better the influence of coating preparation on the alteration of bulk elasticity. Figure 1 illustrates an overview of the applied hydrogel coating method and the main parameters investigated that affect the coating and bulk PDMS.

## 2. Experimental

### 2.1. Materials 

The polydimethylsiloxane (PDMS) was prepared using a Sylgard 184 elastomer kit acquired from Dow Corning, VWR chemicals, Amsterdam, The Netherlands, and the preparation was done according to the supplier’s information. Benzophenone (BP), *N*-Isopropylacrylamide (NIPAM), and 2-Hydroxyethyl methacrylate (HEMA) from Sigma-Aldrich, Zwijndrecht, The Netherlands; acetone from VWR chemicals, Amsterdam, The Netherlands; and acrylamide (AAm) from LKB Bromma, Mariehäll, Sweden were used as received. The surface preparation of PDMS was conducted in a nitrogen-filled glove box.

### 2.2. Modification of PDMS Substrates

PDMS samples were prepared by mixing the silicone elastomer base and silicone curing agent at a weight ratio of 10:1. To prevent bubble formation, the mixture was degassed under vacuum. Two grams of the mixture was poured into a 2 × 0.7 cm mold, providing a 1 mm thick PDMS substrate. Molds were placed in an oven and cured at 70 °C overnight. UV-mediated free radical polymerization onto the PDMS was performed based on previous approaches, with some adjustments [12,34] to optimize the coating procedure. All solutions used for coating preparation were degassed by purging with nitrogen for 60 min prior to use. The PDMS substrates were incubated in a benzophenone solution in acetone (10 wt %) for 15 min under a nitrogen atmosphere and dried. The benzophenone-infused PDMS substrate was placed in a quartz cuvette. Aqueous monomer solutions with either *N*-Isopropylacrylamide (NIPAM) 10 wt %, 2-Hydroxyethyl methacrylate (HEMA) 20 wt %, or acrylamide (AAm) 1 wt %, 5 wt %, 10 wt %, 20 wt % were prepared in ultrapure water. The degassed monomer solution was then loaded into the quartz cuvette, sealed with the PDMS substrate, and subsequently irradiated by UV using a Spectrolinker XL 1500 UV source (Spectronics Corp., Westbury, NY, USA) with eight, fluorescent, 15-W black light tubes; the UV (F15T8/BLB GTE Sylvania) light was predominantly at a wavelength of 365 nm. The UV lamp provided an intensity between 2300–1100 µW/cm^2^. After the polymerization, the coated PDMS substrates were washed in 20 mL ethanol for 1 h to remove the non-reacted compounds, followed by washing in 20 mL water for 1 h at room temperature. The samples were dried under a nitrogen atmosphere at room temperature for 24 h.

### 2.3. Instrumentation

**Water Contact Angle:** The degree of surface modification and wettability properties of the surfaces were determined by measuring the static water contact angles (WCA) over time at room temperature. Droplets were placed on the surface with a syringe (1–1.5 µL), and contact angles were measured using a homemade contour monitor over a period of 600 s. Control measurements were performed to identify if potential evaporation affected the measurements, which was not the case. 

**Atomic Force Microscopy:** The surface morphology of the coatings was imaged using an atomic force microscopy (AFM) model Dimension 3100 Nanoscope V system (Veeco, Plainview, NY, USA) in contact mode and with 0.24 N/m tips. All data were processed using Nanoscope Analysis (Veeco, Version 1.70).

**Scanning Electron Microscopy and Energy-dispersive X-ray Spectroscopy:** The cross-section of the coatings was examined using a Philips ESEM-XL30 scanning electron microscope (SEM; SEMTech solutions, North Billerica, MA, USA) equipped with a field emission gun operating at 20 kV. Prior to SEM examination, the specimens were prepared by freeze-fracturing after immersion in liquid N_2_ and coated with gold. The elemental composition was determined using energy-dispersive X-ray spectroscopy (EDS) operating at an accelerating voltage of 15 kV. Due to the poor contrast in the SEM observation between the polymer coatings and PDMS substrate, the thickness of polymer coatings was measured based on the composition profile of the tracing element on fractured cross sections of the coated PDMS samples using SEM imaging and EDS line scanning. The measurement was performed at 3 different locations on each fractured sample and then averaged.

**Determining Young’s modulus:** The stiffness (Young’s modulus) of the coated PDMS samples was tested using a Zwick Z 2.5 universal testing machine (Zwick/Roell, Ulm, Germany). For the measurement, dumbbell shaped specimens with 1 mm thickness were prepared. The wider end-sections of these samples were clamped into the testing device and the narrower gauge region was investigated. Uniaxial tension was applied to measure the strain and stress. A loading speed of 1 mm/min was applied until fracture. All measurements were performed in triplicate. Acquired values for stress and strain were extrapolated using Hooke’s Law, E = σ/ε, where σ is the applied stress and ε is the resultant strain. The Young’s modulus was calculated by linear regression using the data from the stress–strain region below 5% elongation.

## 3. Results and Discussion

UV-mediated free radical polymerization, with surface-infused benzophenone as an initiator, was successfully carried out on PDMS surfaces under nitrogen atmosphere. In this study NIPAM, HEMA, and AAm were selected as model monomers for the surface modification. Upon irradiation, benzophenone generates free radicals on the surface, thereby absorbing H-atoms from the methyl groups of PDMS. Subsequently, the free radicals engage in free radical polymerization reactions, providing covalently-bound polymer coatings. We chose AAm as the monomer for investigating the polymerization under various reaction conditions, such as with different UV irradiation times (5 min, 15 min, 30 min, and 60 min) and four monomer concentrations (1 wt %, 5 wt %, 10 wt %, and 20 wt %). The optimum reaction conditions for coating formation while maintaining bulk properties were identified by applying this systematic approach and investigating the coating and bulk properties via various techniques.

### 3.1. Monomer Type, Concentration, and UV-Irradiation Time Affect Coating Characteristics

**Figure 2:**
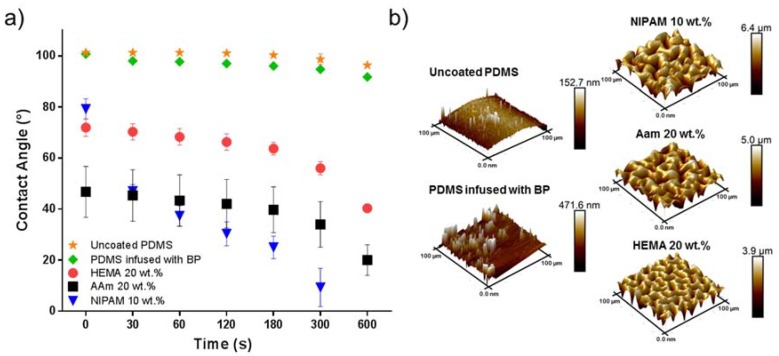
(**a**) Contact angle measurements as a function of time and (**b**) surface morphology images captured by AFM of an uncoated PDMS surface; PDMS infused with benzophenone (BP); and PDMS surface coated with *N*-Isopropylacrylamide (NIPAM) 10 wt %, 2-Hydroxyethyl methacrylate (HEMA) 20 wt %, and acrylamide (AAm) 20 wt % (UV irradiation time is 10 min).

To study the effect of UV irradiation time, monomer type, and concentration on the degree of coating formation, WCA characterizations were performed. The influence of parameter variations on surface morphology of the coatings were investigated by AFM. Figure 2a shows WCA measurements for 1–1.5 µL water droplets placed onto the surface of the uncoated PDMS; benzophenone infused PDMS; and PDMS coated with 10 wt % NIPAM, 20 wt % HEMA, and 20 wt % acrylamide after 10 min UV irradiation. As illustrated, the uncoated PDMS was hydrophobic, with a WCA of approximately 100°. In addition, the PDMS infused with benzophenone displayed similar hydrophobicity. The WCA measurements clearly indicate that the PDMS surfaces were becoming hydrophilic after modification with these three monomers. Figure 2b shows the AFM analysis of the surface morphology of uncoated PDMS; BP infused PDMS; and PDMS coated with 10 wt % NIPAM, 20 wt % HEMA, and 20 wt % acrylamide after 10 min of UV radiation. Rough surface structures were observed after the modifications with different monomers compared to the unmodified PDMS and PDMS infused with BP. The presence of the coatings was also apparent because the transparent PDMS surface became opaque after the coating was formed. All modifications with monomers resulted in hydrophilic surfaces due to the hydrophilic nature of the monomers used. The final WCAs were 40°, 20°, and 10° for HEMA, AAm, and NIPAM, respectively It is noteworthy that the WCA of coated PDMS decreased over time. Although all three coatings were hydrophilic in the end, the time to reach hydrophilicity was different, which reflects the reorganization speed of the polymer network inside the hydrogel coatings. As the coatings were dried, a specific organization occurred at the interface to minimize interfacial tension. Hydration of this layer during WCA measurements initiated the rearrangements of the polymers to accommodate the change in environment polarity.

**Figure 3:**
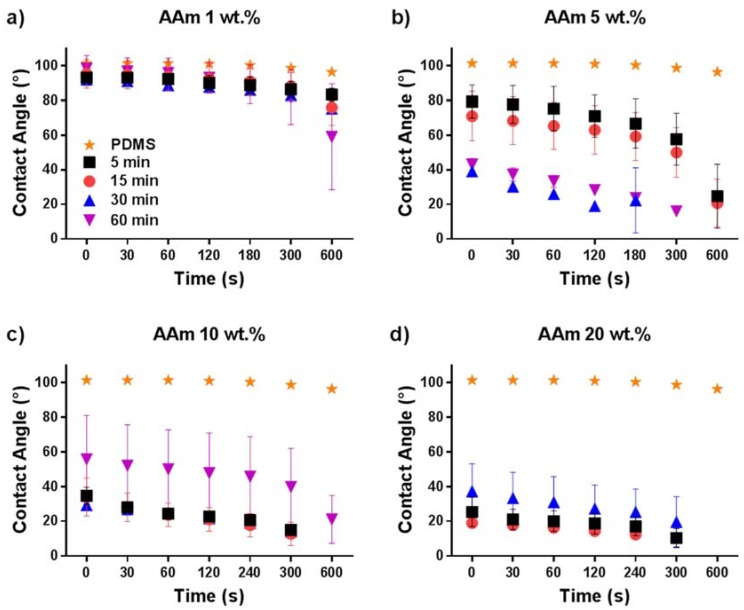
Contact angle measurements of PDMS surfaces coated with different concentrations of acrylamide and with various UV irradiation times: (**a**) AAm 1 wt %, (**b**) AAm 5 wt %, (**c**) AAm 10 wt % and (**d**) AAm 20 wt %.

**Figure 4:**
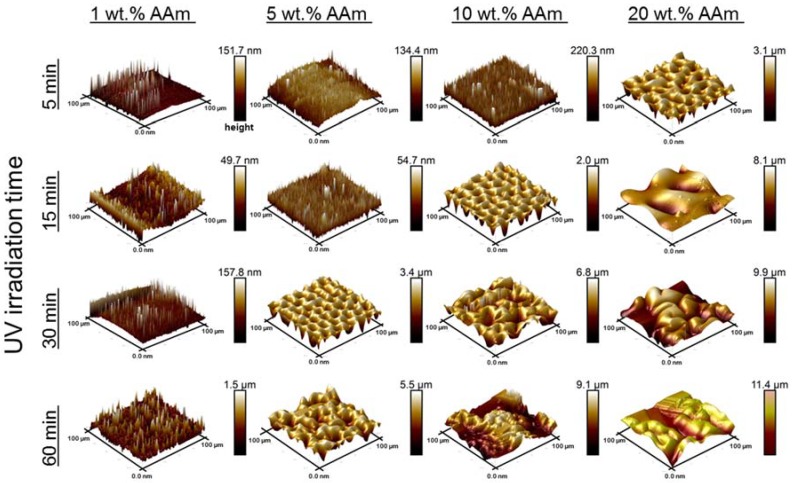
Surface morphology images captured by AFM of representative PDMS surfaces coated with different concentrations of acrylamide and with various UV irradiation times: AAm 1 wt %, AAm 5 wt %, AAm 10 wt %, and AAm 20 wt %. Areas analyzed are 100 µm × 100 µm.

Hydrophilic polymer coatings on PDMS lead to enhanced hydrophilicity, and the speed of surface hydration differs by used monomers. To obtain further insights on the wetting behavior of the coatings formed on the PDMS surface, a single monomer type was used and the monomer concentration and UV-irradiation time were varied. Again, WCA measurements and surface morphology analysis by AFM were performed. Coating preparations were done using AAm with different concentrations (1 wt %, 5 wt %, 10 wt % and 20 wt %) and various UV irradiation times (5 min, 15 min, 30 min, and 60 min). In Figure 3, the WCAs are shown for all these conditions. It can be seen in Figure 3a that varying the UV radiation time from 5 to 60 min for the PDMS modified with 1 wt % AAm had only a mild impact on the measured WCA, which was reduced to 70° for irradiation times of 5–30 min and 60° for 60 min irradiation. This mild change of WCA may be due to the very low AAm concentration, which was not enough to grow a proper polymer layer on the surface because the termination of the polymerization becomes more dominant with respect to propagation. The slight change in WCA was also reflected by the surface morphology as shown in Figure 4. AFM images in the left column show that the modification with 1 wt % AAm did not affect the surface morphology and roughness, irrespective of the UV irradiation time. Most likely, there was an incomplete surface coverage of polymer coating because the monomer concentration was too low. The same effect was also observed for the 5 wt % AAm modification after UV polymerization of 5 and 15 min, respectively, according to the AFM images (second column, Figure 4) and WCA measurements (Figure 3b). The surface morphology of these coatings was not as developed as the coatings obtained with higher concentration of AAm, as shown in Figure 4, even though they seemed more homogenous. The homogeneity of the coating is reflected by the WCA, which decreased over time but not as rapidly as those coatings from higher AAm concentrations and with similar irradiation times, or with the same concentration of AAm but longer irradiation times. Longer UV irradiation time, 30 and 60 min, did show the more strongly hydrophilic character. The WCA values were less than 45° in both cases and the rougher surface morphology, as shown by AFM, supports this notion. These findings show the degree of modification on the surface was better for these conditions when compared to 5 and 15 min irradiated coatings. 

The larger decrease in WCA when the AAm concentration was 10 wt % with 5, 15, and 30 min irradiation times represents the degree of coating formation (Figure 3c). As clearly seen in Figure 4, the polymer layer obtained after 5 min irradiation was not as rough as those obtained after 15 and 30 min of irradiation. This indicates that the AAm concentration (10 wt %) was sufficient to form a proper polymer layer on the surface with 5 min UV irradiation, although the surface is not as rough as when longer UV irradiation times are used. A similar decrease can be seen in the contact angle results for the samples that were modified for 5, 15, and 30 min UV irradiation with 20 wt % of AAm. However, as seen in Figure 4, the surface morphology was quite inhomogeneous for the 10 wt % AAm and 60 min UV reaction as well as 20 wt % AAm and 30 min UV reaction. The reason why the WCA values have a high standard deviation for these samples may be the inhomogeneity of the coating. WCA measurement was not possible for the modified sample with 20 wt % AAm and 60 min UV irradiation time because the surface was not flat; higher monomer concentration and long UV irradiation time led to severe substrate deformations. It should be noted that the surface structures reflect the dry state and that after hydration, these features are less prominent due to swelling of the hydrogel layer.

**Figure 5:**
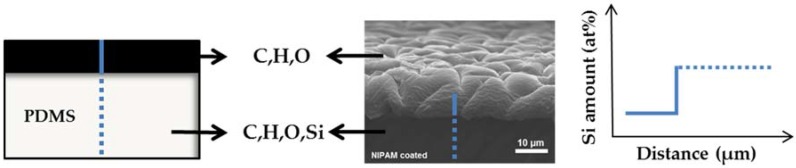
Scheme of Si amount determination from a cross section image of a coated PDMS surface obtained by SEM-EDX.

To measure the coating thickness, cross-sectional EDS line scans were performed on freeze-fractured coated PDMS samples. In our case, PDMS itself consists of Si, C, H and O, while the polymer coating does not contain Si. From this point of view, the composition profile of Si on fractured cross-sections can be used to measure the thickness of the polymer coating (Figure 5). A representative graph can be found in the Supplementary Information (Figure S1). This method was used as an alternative to AFM analysis because the conventional removal of coating from the surface by scratching with a cannula proved unsuccessful. In addition, attempts to perform site-specific modifications with subsequent scanning of the interface between the modified and unmodified PDMS did not function correctly, as the area between modified and unmodified was not sharp enough, resulting in the scanning area being too large for proper AFM height profiling. 

**Figure 6:**
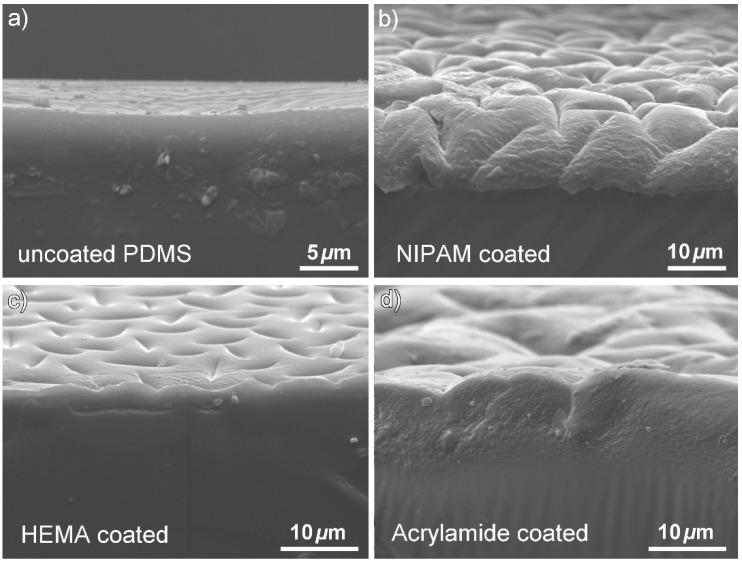
SEM cross section images: (**a**) uncoated PDMS surface, (**b**) PDMS surface coated with NIPAM 10 wt %, (**c**) AAm 20 wt %, and (**d**) HEMA 20 wt %.

In Figure 6, it can be seen from the SEM cross section images that the NIPAM, HEMA, and AAm modifications resulted in different coating thicknesses and morphology. This difference may be due to the different reaction kinetics of these monomers. In radical polymerization, the acrylate group of NIPAM and AAm, on which propagation step occurs, will have a secondary free radical. However, the methacrylate-propagating group of HEMA will carry a tertiary free radical. Tertiary radicals are more stable than secondary radicals. Therefore, the polymerization rate would be slower for methacrylate derivatives, which directly affects the polymer layer formation. It can be concluded that the thickness of the polymer layer is connected to the type of monomers used to modify the PDMS substrate. In addition, we have demonstrated that it is possible to control the coating thickness by the using UV-mediated free radical polymerization approach. In this case, the polymer layer thickness can be easily regulated by varying the acrylamide concentration and UV irradiation time.

**Figure 7:**
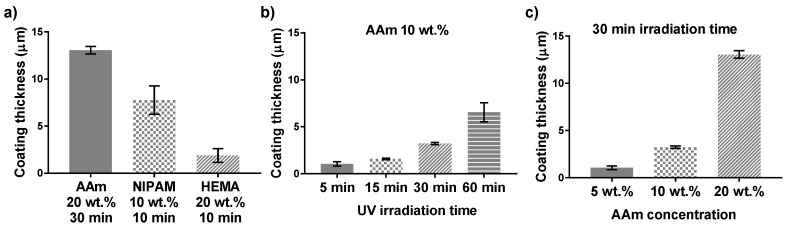
Coating thicknesses of coated PDMS surfaces according to Si content profile by SEM-EDS: (**a**) PDMS surface coated with acrylamide 20 wt %, NIPAM 10 wt %, and HEMA 20 wt %, irradiated for 30, 10, and 10 min, respectively; (**b**) PDMS surface coated with acrylamide 10 wt % and irradiated with different UV irradiation times; (**c**) PDMS surface coated with different concentrations of acrylamide and UV irradiated for 30 min.

The relation between coating thickness and UV irradiation time for the 10 wt % acrylamide coated samples is shown in Figure 7b. The error bars represent the standard deviation of the three different positions along the cross section of the sample. It is observed that the coating thickness increased with an increase with increasing UV irradiation time. On the other hand, with 30 min UV polymerization time and higher monomer concentration resulted in thicker polymer layers (Figure 7c). A higher AAm concentration and longer UV irradiation times resulted thicker coatings, which may be associated to the final polymer density. 

### 3.2. Bulk Material Properties Are Affected by Coating Procedures

**Figure 8:**
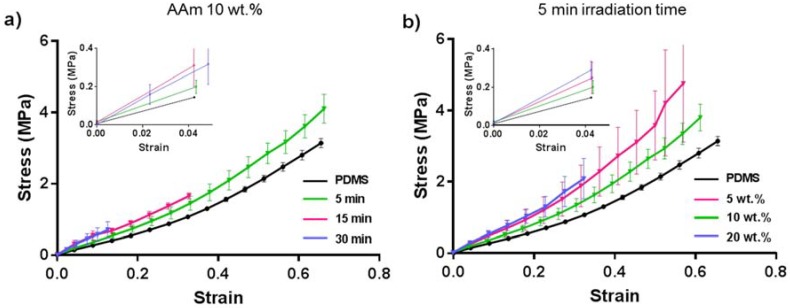
Stress-strain diagram of (**a**) PDMS and PDMS coated with 10 wt % acrylamide irradiated for different UV radiation time, (**b**) PDMS and PDMS coated with acrylamide of different concentrations after 5 min UV radiation.

It was observed that for some specific reaction conditions, the bulk PDMS became brittle and deformed after applying the coating. Therefore, we performed tensile tests (Figure 8) to understand the mechanical properties of the bulk material depending on the applied coating conditions, with coated PDMS samples of 10 wt % AAm with 5, 15, and 30 min UV irradiation. The Young’s modulus of the coated samples was calculated from the linear elastic region (<5% strain of the stress–strain curves (Figure 8) using Hooke’s law, and the results are summarized in Table 1.

The Young’s modulus of the specimens was found to increase with the UV irradiation time for the 10 wt % acrylamide coated samples. The Young’s modulus of the specimen with 5 min irradiation at 10 wt % (4.9 ± 0.5 MPa), was slightly higher than unmodified PDMS, 3.7 ± 0.1 MPa. The Young’s modulus of the 15 and 30 min specimen were even higher (8.2 ± 3 MPa and 6.2 ± 3 MPa, respectively; Table 1). Although, the average value for the Young’s modulus for the 15 and 30 min specimens appeared different, due to the large standard deviation, according to statistical analysis (one-way ANOVA), these are the same (*p* = 0.249, significant difference when *p* < 0.05). 

The elongation-until-break values (%) showed an inverse tendency of the specimen to break with respect to the Young’s Modulus. When the material stiffness increased, the bulk became more brittle causing earlier failure during the elongation test (Figure 8a). It was not possible to perform the elongation test for the sample coated with 10 wt % AAm and 60 min UV-irradiation time, as the PDMS was heavily affected and readily broke while being placed into the measuring setup.

To investigate the influence of monomer concentration on tensile strength, tensile tests were done with the samples coated using 5 min UV irradiation and 5 wt %, 10 wt %, and 20 wt % AAm solution. It can be seen in Figure 8b that with the highest (20 wt %) AAm concentration, the originally soft bulk PDMS turned more rigid and brittle. This coated sample could elongate 34% until breaking, and the Young’s modulus was higher than that of PDMS (Table 1). However, there was no significant difference on the brittleness/softness and elongation properties of the samples that were coated with 5 and 10 wt % acrylamide and irradiated with UV for 5 min. Apparently, long UV polymerization time and high monomer concentration affected the bulk material properties, making PDMS brittle after the coating procedure. It has been reported that long-term exposure of PDMS to UV irradiation affects its properties and that scissoring of side-chains leads to oxidation, which could explain the more brittle nature of the material [44]. This may be an essential problem for many applications including insulating coatings, microfluidics, biomedical devices, and implants, as the material property and the softness of the original PDMS must be maintained. Therefore, preservation of the material bulk property should be considered when enhancing the surface properties of PDMS by polymer coatings for its applications.

## 4. Discussion

On many occasions, the approach of benzophenone-infused PDMS has been used to perform surface modifications and to create hydrogel layers [34,35,36,37,38]. However, it has not been reported that the reaction conditions, such as the presence of monomer, UV irradiation, solvent, etc., negatively affect the bulk properties of the elastomer. It may well be that such extensive alterations to the bulk materials have not been reported as it could have been overlooked depending on the applied conditions. On various occasions, the benzophenone-infused PDMS approach is used to fuse a predefined hydrogel layer onto the PDMS surface or the benzophenone is used as additional cross-linker for pre-coated surfaces [36,45]. Hence, there is no monomer present during the UV-irradiation. Additionally, the polymerizations are often performed in thin films rather than in bulk solution. Using small volumes, in either microchannels or liquid layers between the substrate and a cover glass, enhances reactivity and even though the overall monomer concentration is high, in absolute terms it is minor [46,47]. The enhanced reactivity reduces the UV exposure time. In many of the studies, the UV irradiation time is substantially shorter because activation has been reported to be in the order of seconds and minutes [48]. Avoiding larger volumes and long UV exposure seem to be key aspects to maintain bulk properties. Even in the past, a system highly similar to that presented here also used of long UV-irradiation times (120 min) in the presence of monomer for the preparation of zwitterionic polymer coatings on PDMS via thin liquid films [49]. The main differences in the study by Goda and co-workers were that in addition to the very long UV exposure times, pretreatment was done on the PDMS using plasma oxidation. Plasma oxidation prevents many of the methyl-groups from being activated by benzophenone but also makes the surface denser and less penetrable for small molecules. The pretreatment also impacted on the layer thickness, which was below 100 nm. Therefore, this reduced penetrability seems also to be a key aspect for maintaining bulk properties because no differences in bulk mechanical properties were observed. It is likely that a specific combination of factors will affect the bulk material properties, in particular the presence of bulk monomer solution. However, when aiming for extremely thick and mechanically robust coatings, such bulk solution approaches need to be taken into account.

## 5. Conclusions

Acrylamide monomer coatings on PDMS substrates via UV mediated free radical polymerization using benzophenone were successfully modulated by changing the acrylamide monomer concentration and controlling the UV irradiation time. Altering the UV polymerization time and the monomer concentration significantly affected the wettability performance and the morphology of the surface, as well as the thickness of the hydrogel coatings. Increasing the monomer concentration and UV reaction time resulted in more hydrophilic, rough surfaces, and thicker polymer coatings. Additionally, we demonstrated that the reaction conditions affected not only the surface but also bulk material properties, illustrating the importance of connecting surface properties and bulk material characteristic for optimal functional PDMS elastomeric materials. Additionally, this study revealed that the monomer type used for hydrogel coating has an impact on the surface properties. The reaction kinetics of the polymerization reaction differs between the various monomers. The coatings rendered the surface more hydrophilic compared to uncoated PDMS, which could enhance the PDMS surface characteristics. These insights provide the tools for optimizing hydrogel coatings on elastomeric materials, such as PDMS, and indicate the importance of assessing the full composition of the materials including coating and bulk material. 

## Figures and Tables

**Table 1:** Young’s modulus and elongation percentage values at the breaking point of uncoated and coated PDMS samples.

Sample	Young’s Modulus (MPa)	Elongation at Break (%)
PDMS	3.7 ± 0.1	76.0 ± 9
10 wt %—5 min	4.9 ± 0.5	68.2 ± 13
10 wt %—15 min	8.2 ± 3 *	34.0 ± 5
10 wt %—30 min	6.2 ± 3 *	22.2 ± 21
10 wt %—60 min ^A^	-	-
5 wt %—5 min	6.1 ± 2 ^#^	65.2 ± 7
10 wt %—5 min	4.9 ± 0.5 ^#^	68.2 ± 13
20 wt %—5 min	7.2 ± 1	34.0 ± 6

^A^ measuring this condition was unsuccessful due to highly brittle material. * Statistical analysis indicates no difference between the two conditions according to one-way ANOVA (*p* = 0.249; *p* < 0.05 depicts significance). ^#^ Statistical analysis indicates no difference between the two conditions according to one-way ANOVA (*p* = 0.184; *p* < 0.05 depicts significance).

## Supplementary Materials

The following are available online at http://www.mdpi.com/2073-4360/10/5/534/s1, Figure S1: Si (at %) amount of uncoated PDMS surface and PDMS surface coated with NIPAM 10 wt % as a function of distance. Data extracted from SEM-EDX measurement.
