# Supplementary material for: The Relationship between Bulk Silicone and Benzophenone-Initiated Hydrogel Coating Properties
Source: Polymers (Basel). 2018 May 16;10(5):534. doi: 10.3390/polym10050534 (PMC6415430; doi:10.3390/polym10050534)
Supplement: Supplementary file 1 [file polymers-10-00534-s001.pdf]

Supplementary Materials:

The relationship between bulk silicone and benzophenone-initiated hydrogel coating properties

Damla Keskin [1], Taraneh Mokabbar [2], Yutao Pei [2], Patrick van Rijn [1,3,*]

[1] University of Groningen, University Medical Center Groningen, Department of Biomedical Engineering-FB40, W.J. Kolff Institute for Biomedical Engineering and Materials Science-FB41, A. Deusinglaan 1, 9713 AV Groningen, The Netherlands; d.keskin@umcg.nl (D.K); : p.van.rijn@umcg.nl (P.R.)

[2] University of Groningen, Engineering and Technology Institute Groningen, Nijenborgh 4, 9747 AG Groningen, The Netherlands; t.mokabber@rug.nl (T.M.); y.pei@rug.nl (Y.P.)

[3] University of Groningen, Zernike Institute for Advanced Materials, Nijenborgh 4, 9747 AG Groningen, The Netherlands

* Correspondence: p.van.rijn@umcg.nl (P.R.); Tel.: +31-50-361-6066

CONTENTS:

S1 Si (at%) amount as a function of distance

S1 Si (at%) amount as a function of distance

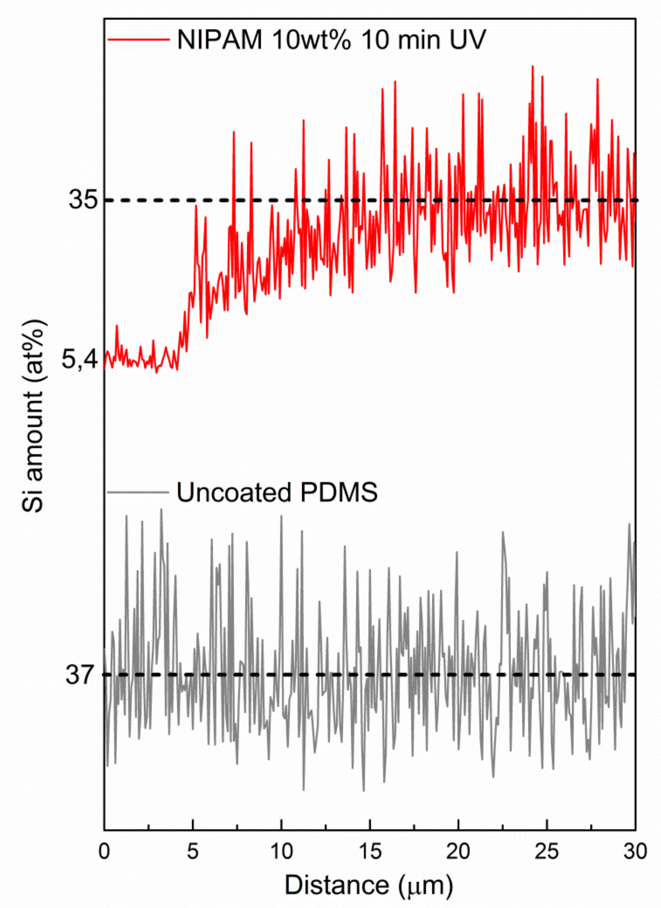

Figure S1: Si (at%) amount of uncoated PDMS surface and PDMS surface coated with NIPAM 10 wt% as a function of distance. Data extracted from SEM – EDX measurement.
